# Publisher Correction: Deconstructing and repurposing the light-regulated interplay between *Arabidopsis* phytochromes and interacting factors

**DOI:** 10.1038/s42003-019-0726-6

**Published:** 2019-12-17

**Authors:** David Golonka, Patrick Fischbach, Siddhartha G. Jena, Julius R. W. Kleeberg, Lars-Oliver Essen, Jared E. Toettcher, Matias D. Zurbriggen, Andreas Möglich

**Affiliations:** 1 Lehrstuhl für Biochemie, Universität Bayreuth, 95447 Bayreuth, Germany; 2 Institute of Synthetic Biology and CEPLAS, Heinrich Heine University Düsseldorf, 40225 Düsseldorf, Germany; 3 Department of Molecular Biology, Princeton University, Princeton, NJ 08544 USA; 4 Department of Chemistry, Center for Synthetic Microbiology, Philipps University Marburg, 35032 Marburg, Germany; 5 Research Center for Bio-Macromolecules, Universität Bayreuth, 95447 Bayreuth, Germany; 6 Bayreuth Center for Biochemistry & Molecular Biology, Universität Bayreuth, 95447 Bayreuth, Germany; 7 North-Bavarian NMR Center, Universität Bayreuth, 95447 Bayreuth, Germany

**Keywords:** Proteins, Biophysical methods, Biological fluorescence

Correction to: *Communications Biology* 10.1038/s42003-019-0687-9, published online 2 December 2019.

In the original published version of this article, the label “PIF-EYFP” in Figure 2b was partially obscured and illegible. The error has been corrected in the HTML and PDF versions of the article.

