# Supplementary material for: Deconstructing and repurposing the light-regulated interplay between Arabidopsis phytochromes and interacting factors
Source: Commun Biol. 2019 Dec 2;2:448. doi: 10.1038/s42003-019-0687-9 (PMC6888877; doi:10.1038/s42003-019-0687-9)
Supplement: Supplementary file 1 — Supplementary Information [file 42003_2019_687_MOESM1_ESM.pdf]

Supplementary Information - Golonka *et al.*

Supplementary Figures

Supplementary Figure 1

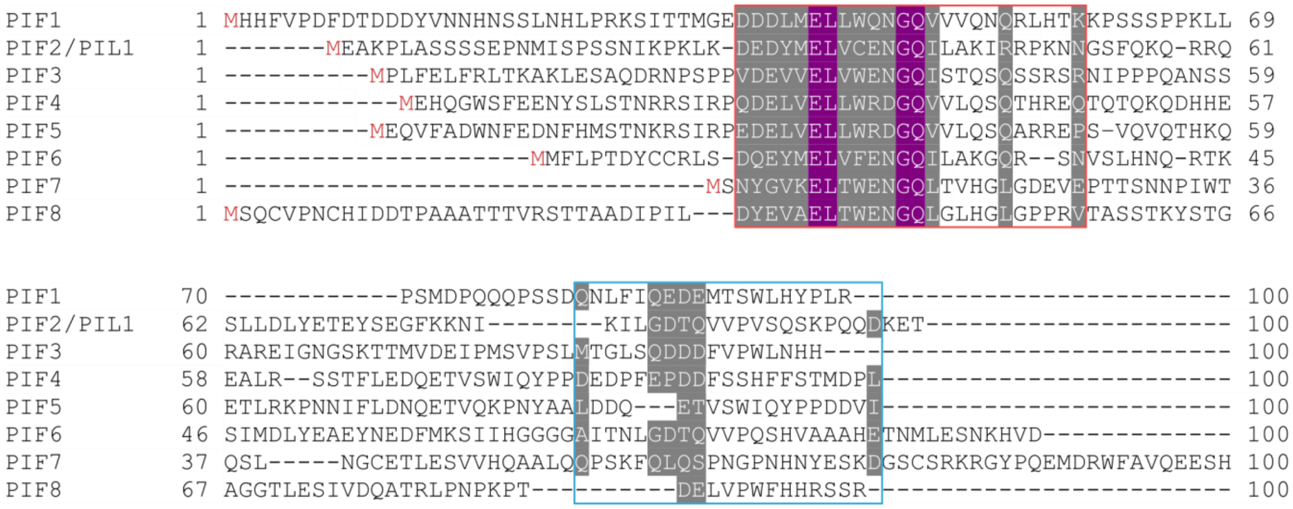

Sequence alignment of the N-terminal segments of the *A. thaliana* PIFs 1-8 according to Khanna *et al.*[1] Red color marks the N-terminal methionine; violet and gray color indicate strictly conserved and moderately conserved residues, respectively. Boxes highlight the conserved APB.A (red) and APB.B (blue) segments.

Supplementary Figure 2

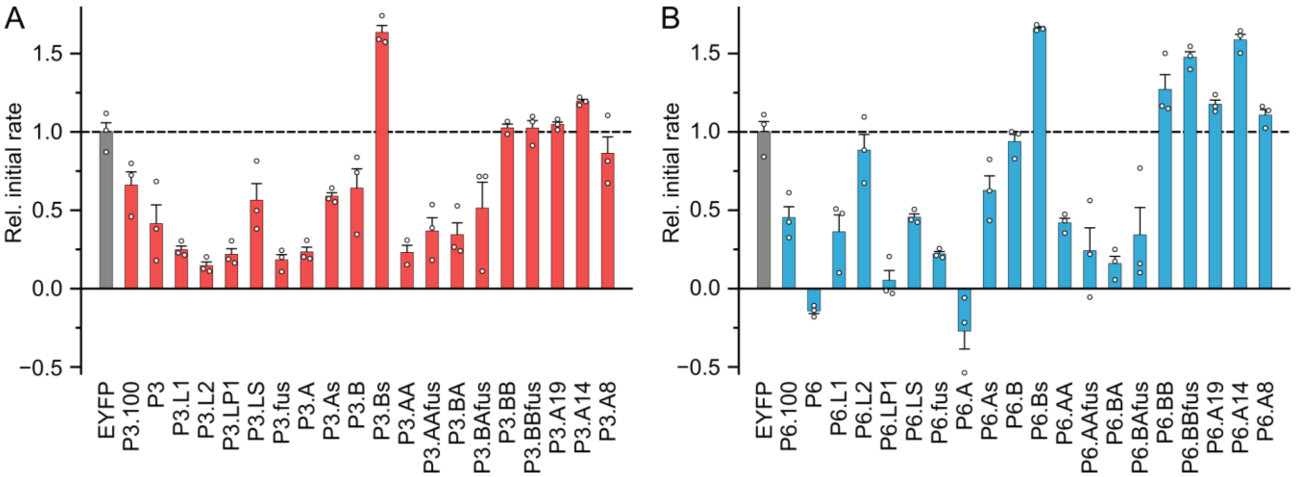

The initial rates of the recovery reaction of the *At*PhyB PCM following red-light exposure were determined in bacterial lysate in the presence of different *At*PIF variants and normalized to the reading obtained for the EYFP negative control. Data indicate mean ± SEM of n = 3 independent biological replicates. See Fig. 2 for details.

Supplementary Figure 3

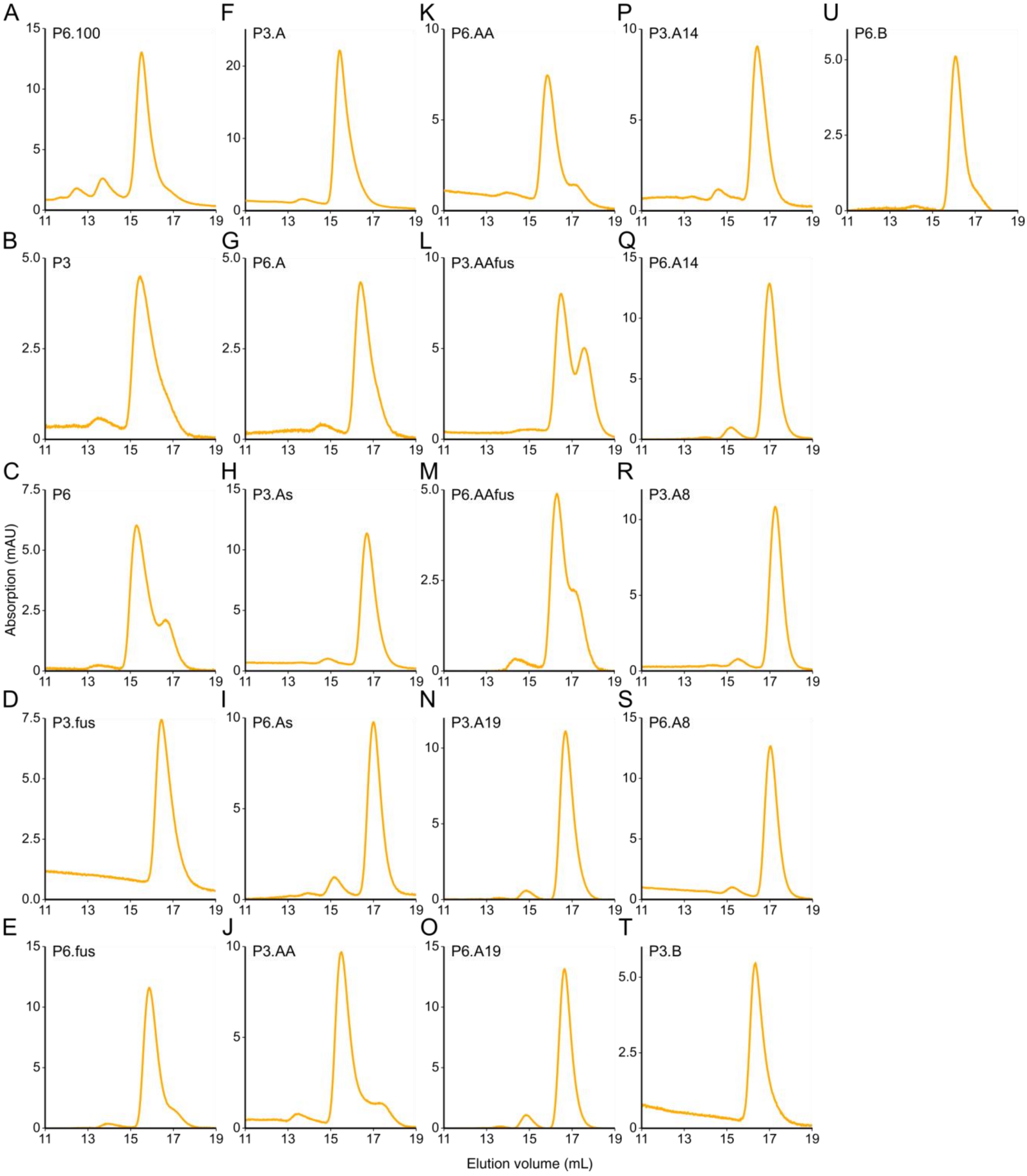

Oligomeric state of the *At*PIF variants. (A) 10 µM P6.100-EYFP were analyzed by size-exclusion chromatography, where the yellow lines represent the absorption at 513 nm. (B-U) As in (A), but for (B) P3; (C) P6; (D) P3.fus; (E); P6.fus; (F) P3.A; (G) P6.A; (H) P3.As; (I) P6.As; (J) P3.AA; (K) P6.AA; (L) P3.AAfus; (M) P6.AAfus; (N) P3.A19; (O) P6.A19; (P) P3.A14; (Q) P6.A14; (R) P3.A8; (S) P6.A8; (T) P3.B; (U) P6.B.

Supplementary Figure 4

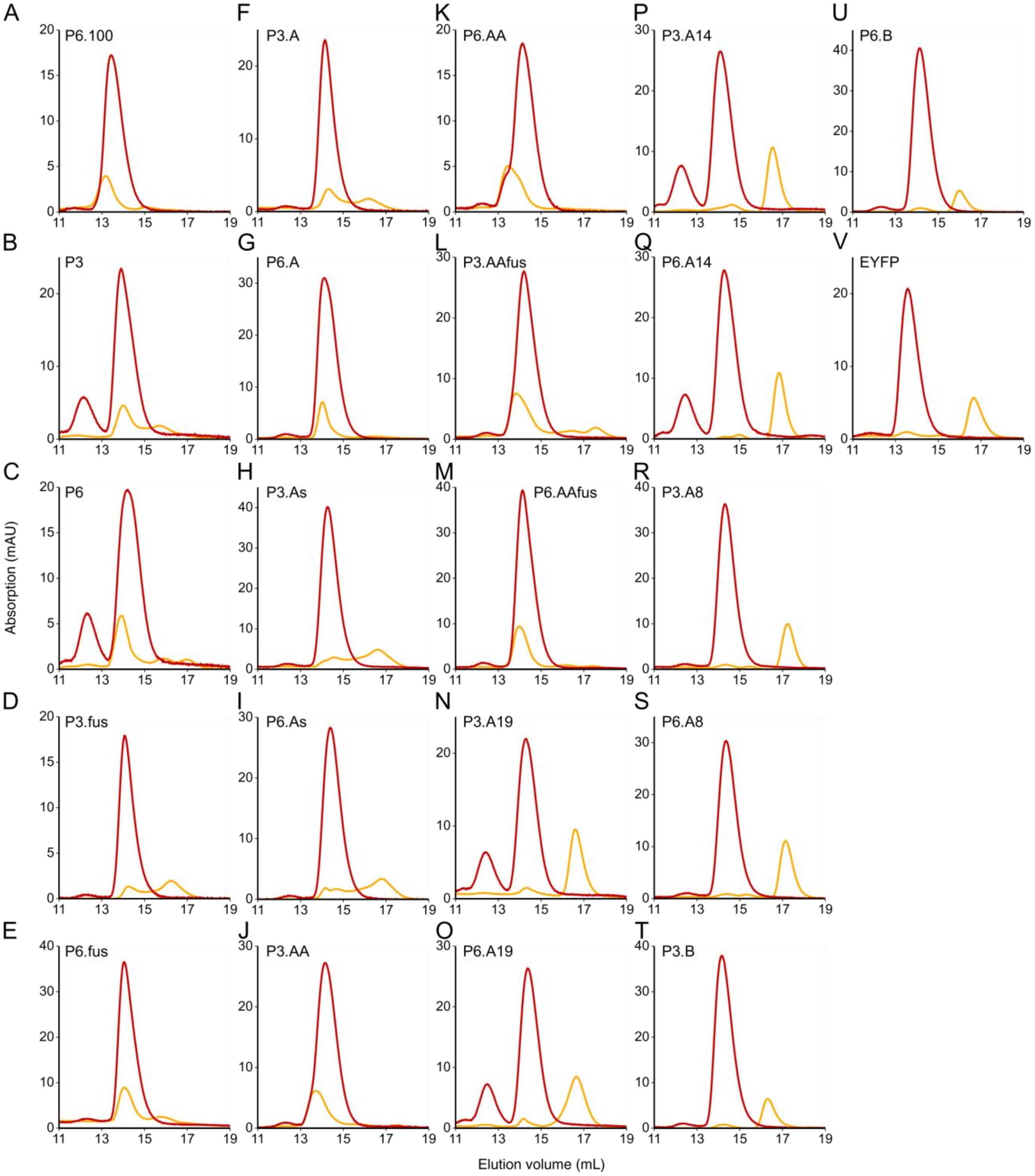

Light-dependent interactions of the *At*PIF variants with the Pfr state of the *At*PhyB PCM. (A) A mixture of 10 µM P6.100-EYFP and 50 µM *At*PhyB PCM was exposed to red light and analyzed by size-exclusion chromatography, where the yellow and red lines represent the absorption at 513 and 650 nm, respectively. (B-V) As in (A), but instead of P6.100-EYFP for (B) P3; (C) P6; (D) P3.fus; (E); P6.fus; (F) P3.A; (G) P6.A; (H) P3.As; (I) P6.As; (J) P3.AA; (K) P6.AA; (L) P3.AAfus; (M) P6.AAfus; (N) P3.A19; (O) P6.A19; (P) P3.A14; (Q) P6.A14; (R) P3.A8; (S) P6.A8; (T) P3.B; (U) P6.B; (V) EYFP. The schematics in the graphs indicate the composition of the *At*PIF variants, with variants deriving from *At*PIF3 and *At*PIF6 shown in red and blue, respectively.

Supplementary Figure 5

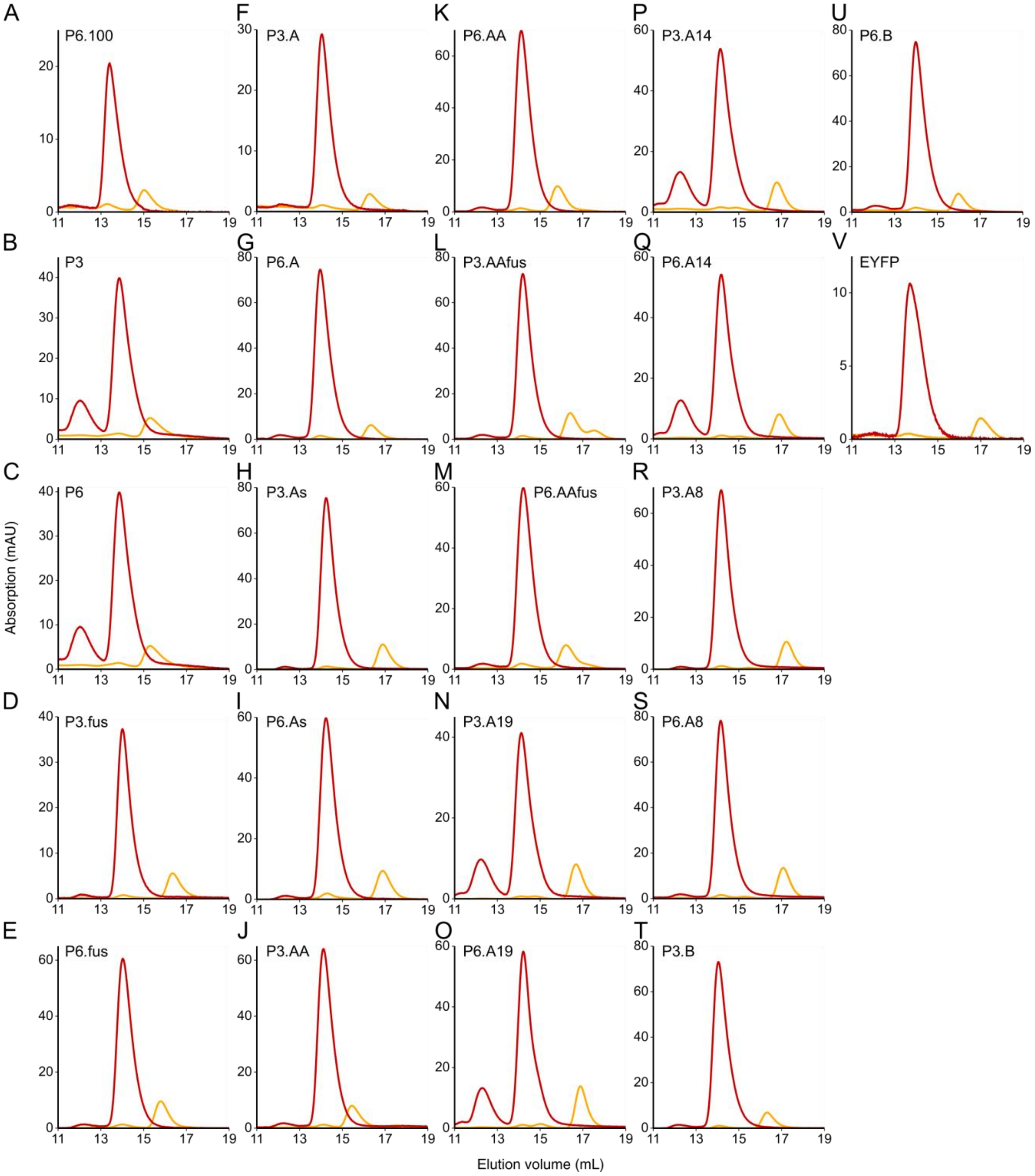

Light-dependent interactions of the *At*PIF variants with the Pr state of the *At*PhyB PCM. (A) A mixture of 10 µM P6.100-EYFP and 50 µM *At*PhyB PCM was exposed to far-red light and analyzed by size-exclusion chromatography, where the yellow and red lines represent the absorption at 513 and 650 nm, respectively. (B-V) As in (A), but instead of P6.100-EYFP for (B) P3; (C) P6; (D) P3.fus; (E); P6.fus; (F) P3.A; (G) P6.A; (H) P3.As; (I) P6.As; (J) P3.AA; (K) P6.AA; (L) P3.AAfus; (M) P6.AAfus; (N) P3.A19; (O) P6.A19; (P) P3.A14; (Q) P6.A14; (R) P3.A8; (S) P6.A8; (T) P3.B; (U) P6.B; (V) EYFP. The schematics in the graphs indicate the composition of the *At*PIF variants, with variants deriving from *At*PIF3 and *At*PIF6 shown in red and blue, respectively.

Supplementary Figure 6

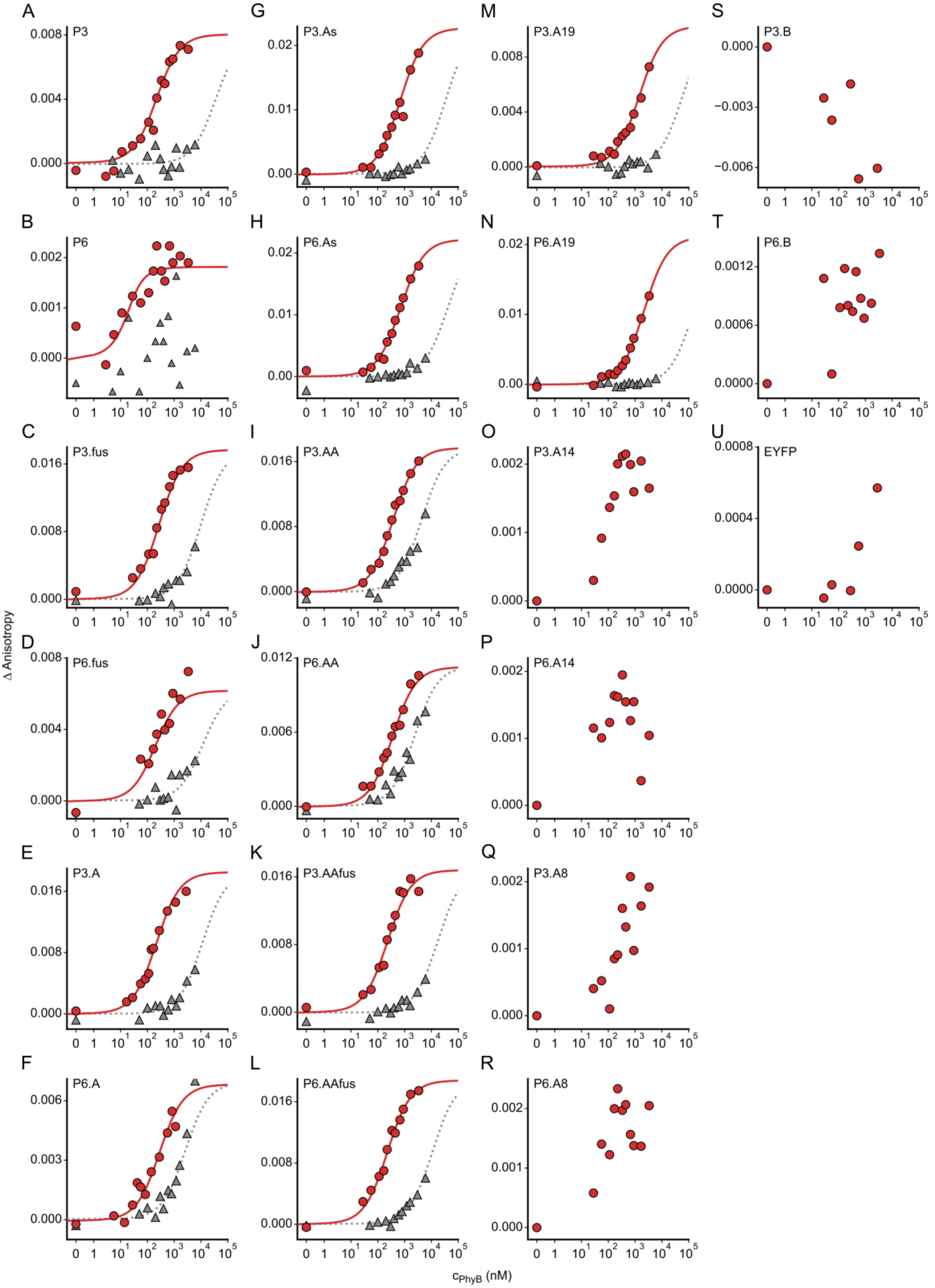

Quantitative analyses of the light-dependent protein:protein interaction between *At*PIF variants and the *At*PhyB PCM. (A) Titration of 20 nM P3-EYFP with increasing concentrations of dark-adapted

(gray) or red-light-exposed *At*PhyB PCM (red), as monitored by anisotropy of the EYFP fluorescence. Data points show averages of three biological replicates. The lines denote fits to single-site binding isotherms. (B-U) As in (A), but instead of P3-EYFP for (B) P6; (C) P3.fus; (D) P6.fus; (E) P3.A; (F) P6.A; (G) P3.As; (H) P6.As; (I) P3.AA; (J) P6.AA; (K) P3.AAfus; (L) P6.AAfus; (M) P3.A19; (N) P6.A19; (O) P3.14; (P) P6.14; (Q) P3.8; (R) P6.8; (S) P3.B; (T) P6.B; (U) EYFP.

Supplementary Figure 7

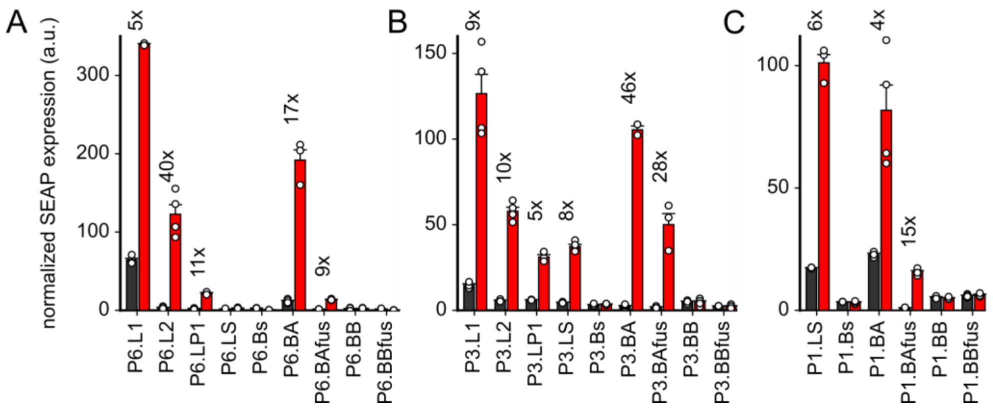

Harnessing the *At*PIF variants for the light-dependent regulation of gene expression in mammalian cells. (A) SEAP expression was determined for the diverse *At*PIF6 variants and normalized to the constitutive expression of *Gaussia* luciferase. Black and red bars denote mean normalized SEAP expression ± SEM for $n = 4$ independent biological replicates under dark conditions or red light, respectively. The numbers above the bars indicate the factor difference between dark and red-light conditions for a given *At*PIF6 variant. (B) As panel (A) but for the *At*PIF3 variants. (C) As panel (A) but for the *At*PIF1 variants.

Supplementary Figure 8

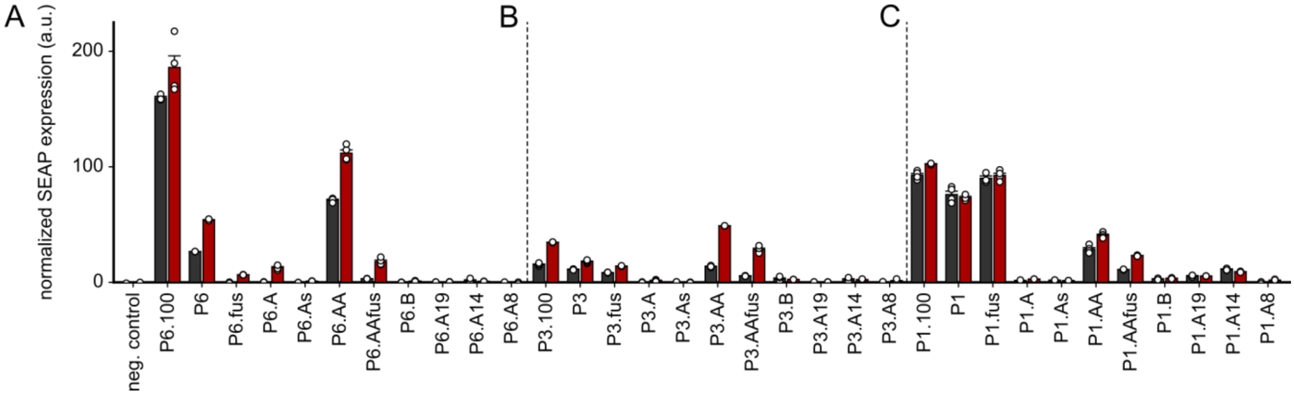

Light-dependent regulation of gene expression in mammalian cells. The experiment was conducted as described in Fig. 5 but the cells were incubated in darkness for 48 h (black bars) or for 24 h under 20 µmol m^{-2} s^{-1} 660-nm light, followed by 20 µmol m^{-2} s^{-1} 740-nm light for another 24 h (brown). (A) SEAP expression was determined for the diverse *At*PIF6 variants and normalized to the constitutive expression of *Gaussia* luciferase. Bars denote mean normalized SEAP expression ± SEM for *n* = 4 independent biological replicates. (B) As panel (A) but for the *At*PIF3 variants. (C) As panel (A) but for the *At*PIF1 variants.

Supplementary Figure 9

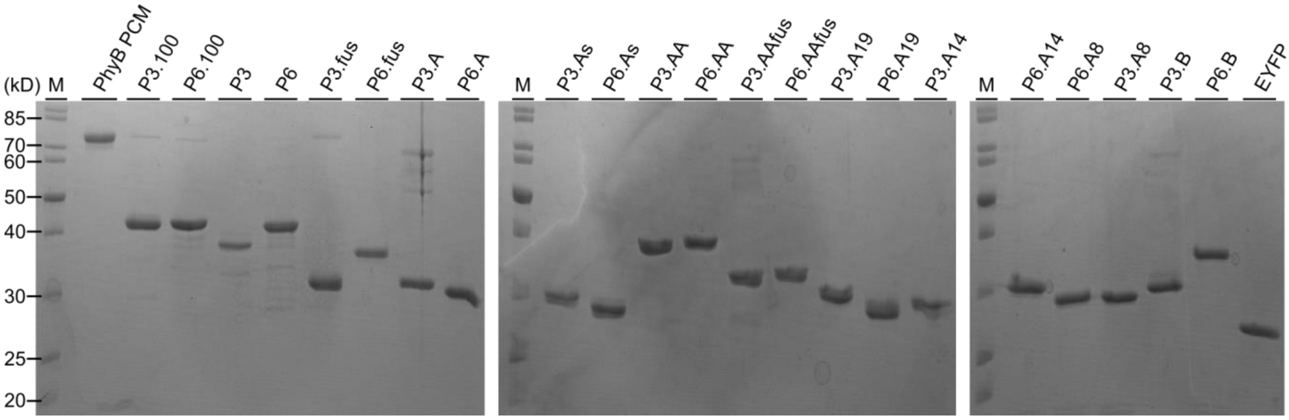

Analysis of the purified *At*PIF3/6-EYFP proteins and the *At*PhyB PCM by denaturing polyacrylamide gel electrophoresis.

Supplementary Tables

Supplementary Table 1. Amino-acid sequences of the *At*PIF variants used in this study.

Name	Sequence[a]
P1.100	MHHFVPDFDT DDDYVNNHNS SLNHLPRKSI TTMGEDDDLM ELLWQNGQVV VQNQRLHTKK PSSSPPKLLP SMDPQQQPSS DQNLFIQEDE MTSWLHYPLR
P3.100	MPLFELFRLT KAKLESAQDR NPSPPVDEVV ELVWENGQIS TQSQSSRSRN IPPPQANSSR AREIGNGSKT TMVDEIPMSV PSLMTGLSQD DDFVPWLNHH
P6.100	MMFLPTDYCC RLSDQEYMEL VFENGQILAK GQRSNVSLHN QRTKSIMDLY EAEYNEDFMK SIIHGGGGAI TNLGDTQVVP QSHVAAAHET NMLESNKHVD
P1	MDDDLMELLW QNGQVVVQNQ RLHTKKPSSS PPKLLPCMDP QQQPSSDQNL FIQEDEMTSW LHYPLR
P3	MVDEVVELVW ENGQISTQSQ SSRSRNIPPP QANSSRAREI GNGSKTTMVD EIPMSVPSLM TGLSQDDDFV PWLNHH
P6	MDQEYMELVF ENGQILAKGQ RSNVSLHNQR TKSIMDLYEA EYNEDFMKSI IHGGGGAITN LGDTQVVPQS HVAAAHETNM LESNKHVD
P3.L1	MVDEVVELVW ENGQISTQSQ SSRSRRAREI GNGSKTTMVD EIPMSVPSLM TGLSQDDDFV PWLNHH
P6.L1	MDQEYMELVF ENGQILAKGQ RSNMDLYEAE YNEDFMKSII HGGGGAITNL GDTQVVPQSH VAAAHETNML ESNKHVD
P3.L2	MVDEVVELVW ENGQISTQSQ SSRSRNIPPP QANSSRAREI GNGSKTTMTG LSQDDDFVPW LNHH
P6.L2	MDQEYMELVF ENGQILAKGQ RSNVSLHNQR TKSIMDLYEA EYNEDAITNL GDTQVVPQSH VAAAHETNML ESNKHVD
P3.LP1	MVDEVVELVW ENGQISTQSQ SSRSRKPSSS PPKLLPCMDP QQQPSSDMTG LSQDDDFVPW LNHH
P6.LP1	MDQEYMELVF ENGQILAKGQ RSNKPSSSPP KLLPCMDPQQ QPSSDAITNL GDTQVVPQSH VAAAHETNML ESNKHVD
P3.LS	MVDEVVELVW ENGQISTQSQ SSRSRDSAGS AGSAGMTGLS QDDDFVPWLN HH
P6.LS	MDQEYMELVF ENGQILAKGQ RSNDSAGSAG SAGAITNLGD TQVVPQSHVA AAHETNMLES NKHVD
P1.fus	MDDDLMELLW QNGQVVVQNQ RLHTKQNLFI QEDEMTSWLH YPLR
P3.fus	MVDEVVELVW ENGQISTQSQ SSRSRMTGLS QDDDFVPWLN HH
P6.fus	MDQEYMELVF ENGQILAKGQ RSNAITNLGD TQVVPQSHVA AAHETNMLES NKHVD
P1.A	MDDDLMELLW QNGQVVVQNQ RLHTKKPSSS PPKLLP
P3.A	MVDEVVELVW ENGQISTQSQ SSRSRNIPPP QANSSRAREI GN
P6.A	MDQEYMELVF ENGQILAKGQ RSNVSLHNQR TKSIMDLYEA
P1.B	MSMDPQQQPS SDQNLFIQED EMTSWLHYPL R
P3.B	MGSKTTMVDE IPMSVPSLMT GLSQDDDFVP WLNHH
P6.B	MEYNEDFMKS IIHGGGGAIT NLGDTQVVPQ SHVAAAHETN MLESNKHVD
P1.As	MDDDLMELLW QNGQVVVQNQ RLHTK

P3.As	MVDEVVELVW ENGQISTQSQ SSRSR
P6.As	MDQEYMELVF ENGQILAKGQ RSN
P1.Bs	MQNLFIQED EMTSWLHYPL R
P3.Bs	MMT GLSQDDDFVP WLNHH
P6.Bs	MAIT NLGDTQVVPQ SHVAAAHETN MLESNKHVD
P1.AA	MDDDLMELLW QNGQVVVQNQ RLHTKKPSSS PPKLLPCMDP QQQPSSDDDD LMELLWQNGQ VVVQNQRLHT KMTSWLHYPL R
P3.AA	MVDEVVELVW ENGQISTQSQ SSRSRNIPPP QANSSRAREI GNGSKTTMVD EIPMSVPSLV DEVVELVWEN GQISTQSQSS RSRFVPWLNH H
P6.AA	MDQEYMELVF ENGQILAKGQ RSNVSLHNQR TKSIMDLYEA EYNEDFMKSI IHGGGGDQEY MELVFENGQI LAKGQRSNTN MLESNKHVD
P1.AAfus	MDDDLMELLW QNGQVVVQNQ RLHTKDDDLM ELLWQNGQVV VQNQRLHTKM TSWLHYPLR
P3.AAfus	MVDEVVELVW ENGQISTQSQ SSRSRVDEVV ELVWENGQIS TQSQSSRSRF VPWLNHH
P6.AAfus	MDQEYMELVF ENGQILAKGQ RSNDQEYMEL VFENGQILAK GQRSNTNMLE SNKHVD
P1.BB	MQNLFIQEDE KPSSSPPKLL PCMDPQQQPS SDQNLFIQED EMTSWLHYPL R
P3.BB	MMTGLSQDDD NIPPPQANSS RAREIGNGSK TTMVDEIPMS VPSLMTGLSQ DDDFVPWLNH H
P6.BB	MAITNLGDTQ VSLHNQRTKS IMDLYEAEYN EDFMKSIIHG GGGAITNLGD TQVVPQSHVA AAHETNMLES NKHVD
P1.BBfus	MQNLFIQEDE QNLFIQEDEM TSWLHYPLR
P3.BBfus	MMTGLSQDDD MTGLSQDDDF VPWLNHH
P6.BBfus	MAITNLGDTQ AITNLGDTQV VPQSHVAAAH ETNMLESNKH VD
P1.BA	MQNLFIQEDE KPSSSPPKLL PCMDPQQQPS SDDDDLMELL WQNGQVVVQN QRLHTKMTSW LHYPLR
P3.BA	MMTGLSQDDD NIPPPQANSS RAREIGNGSK TTMVDEIPMS VPSLVDEVVE LVWENGQIST QSQSSRSRFV PWLNHH
P6.BA	MAITNLGDTQ VVPQSHVAAA HEVSLHNQRT KSIMDLYEAE YNEDFMKSII HGGGGDQEYM ELVFENGQIL AKGQRSNTNM LESNKHVD
P1.BAfus	MQNLFIQEDE DDDLMELLWQ NGQVVVQNQR LHTKMTSWLH YPLR
P3.BAfus	MMTGLSQDDD VDEVVELVWE NGQISTQSQS SRSRFVPWLN HH
P6.BAfus	MAITNLGDTQ VVPQSHVAAA HEDQEYMELV FENGQILAKG QRSNTNMLES NKHVD
P1.19	MDDDLMELLW QNGQVVVQNQ
P3.19	MVDEVVELVW ENGQISTQSQ
P6.19	MDQEYMELVF ENGQILAKGQ
P1.14	MDDDLMELLW QNGQV
P3.14	MVDEVVELVW ENGQI

P6.14	MDQEYMELVF ENGQI
P1.8	MELLWQNGQ
P3.8	MELVWENGQ
P6.8	MELVFENGQ

[a] Red color marks the N-terminal methionine; violet and gray color indicates strictly conserved and moderately conserved residues, respectively.

Supplementary Reference

1. Khanna, R. et al. A Novel Molecular Recognition Motif Necessary for Targeting Photoactivated Phytochrome Signaling to Specific Basic Helix-Loop-Helix Transcription Factors. *Plant Cell* **16**, 3033–3044 (2004).
