# Supplementary material for: Deconstructing and repurposing the light-regulated interplay between Arabidopsis phytochromes and interacting factors
Source: Commun Biol. 2019 Dec 2;2:448. doi: 10.1038/s42003-019-0687-9 (PMC6888877; doi:10.1038/s42003-019-0687-9)
Supplement: Supplementary file 2 — Description of Additional Supplementary Files [file 42003_2019_687_MOESM2_ESM.docx]

# Description of Additional Supplementary Data - Golonka *et al.*

A Microsoft Excel file with multiple sheets containing the experimental data that underpin figures 2C, 2D, 3A, 3B, 3C, 3D, 3E, 3F, 4B, 4C, 4D, 5B, 5C, 5D, 6F and 6G.
